# Aquaporin Expression and Regulation in Clinical and Experimental Sepsis

**DOI:** 10.3390/ijms25010487

**Published:** 2023-12-29

**Authors:** Nikolaos S. Lotsios, Chrysi Keskinidou, Ioanna Dimopoulou, Anastasia Kotanidou, Stylianos E. Orfanos, Alice G. Vassiliou

**Affiliations:** First Department of Critical Care Medicine & Pulmonary Services, School of Medicine, National and Kapodistrian University of Athens, Evangelismos Hospital, 106 76 Athens, Greece; n.lotsios96@gmail.com (N.S.L.); chrysakes29@gmail.com (C.K.); idimo@otenet.gr (I.D.); akotanid@med.uoa.gr (A.K.); stylianosorfanosuoa@gmail.com (S.E.O.)

**Keywords:** aquaporin, sepsis, acute respiratory distress syndrome, single-nucleotide polymorphisms, lnRNA, miRNA

## Abstract

Sepsis is an inflammatory disorder caused by the host’s dysfunctional response to infection. Septic patients present diverse clinical characteristics, and in the recent years, it has been the main cause of death in intensive care units (ICU). Aquaporins, membrane proteins with a role in water transportation, have been reported to participate in numerous biological processes. Their role in sepsis progression has been studied extensively. This review aims to examine recent literature on aquaporin expression and regulation in clinical sepsis, as well as established experimental models of sepsis. We will present how sepsis affects aquaporin expression at the molecular and protein level. Moreover, we will delve into the importance of aquaporin regulation at transcriptional, post-transcriptional, translational, and post-translational levels in sepsis by presenting data on aquaporin regulation by non-coding RNAs and selected chemical molecules. Finally, we will focus on the importance of aquaporin single-nucleotide polymorphisms in the setting of sepsis.

## 1. Introduction

**Figure 1:**
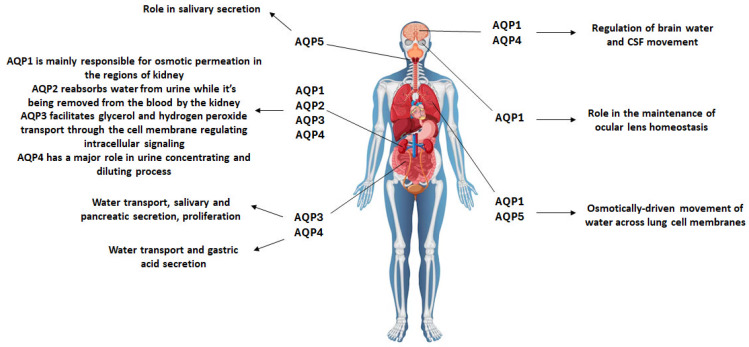
Expression and function of aquaporins. AQP1 is expressed in the brain, kidney, lungs, and eyes; AQP2 is predominantly expressed in the kidneys; AQP3 is expressed in the kidneys, the digestive tract, and immune cells; AQP4 is expressed in the central nervous system, kidneys, and digestive tract; and AQP5 is expressed in the lungs and salivary glands. AQP, aquaporin; CSF, cerebrospinal fluid. This figure has been designed using assets from Freepik.com.

Aquaporins (AQPs) compose a family of membrane proteins whose main function is the regulation of water transportation. To this day, 13 unique mammalian aquaporins have been identified. Several members of the aquaporin protein family are known as aquaglyceroporins and retain a role in the passive transport of glycerol and other small solutes, such as urea and carbon dioxide. AQPs are homo-tetramers consisting of four identical monomers of approximately 30 kDa. These are the molecule’s structural backbone, each having the ability to function as a channel [1]. AQPs are implicated in a plethora of biological functions, which differ among water-selective AQPs and the aquaglyceroporins [2]. Transepithelial fluid, cell migration, and brain edema have been connected to water-selective AQPs. On the other hand, aquaglyceroporins are integral in cell proliferation, adipocyte metabolism, and epidermal water retention. In mammals, aquaporins have been detected primarily in the lung, kidney, eye, and brain, and they have been examined as possible therapeutic targets in disorders characterized by the dysregulation of water homeostasis [3,4]. Most notably, the expression patterns of the aquaporins in mammals are as follows: AQP1: brain, kidney, lungs, and eyes; AQP2: kidneys; AQP3: kidneys, the digestive tract, and immune cells; AQP4: central nervous system, kidneys, and digestive tract; AQP5: lungs and salivary glands; AQP6: renal epithelia; AQP7: liver, kidneys, and cardiac muscle; AQP8: liver, kidneys, and pancreas; AQP9: immune cells; AQP10: gastrointestinal tract; AQP11: endoplasmic reticulum membrane; and AQP12: pancreas, intestine, and stomach [5]. The expression and roles of the various aquaporins under physiological conditions is illustrated in Figure 1.

In recent years, the involvement of AQPs in inflammatory processes has been firmly established [6]. Sepsis is an inflammatory disorder that is defined as life-threatening organ dysfunction caused by the host’s dysregulated response to infection [7]. This disorder’s immense complexity results in high mortality rates. More importantly, in recent years, sepsis has been identified as the primary cause of mortality in intensive care units (ICU) [8]. Furthermore, one of the main difficulties presented by sepsis is the fact that patients’ clinical characteristics do not correlate fully with their outcome. Specifically, patients die at every stage, regardless of their clinical characteristics [9]. 

The aim of this review is to provide a clear understanding of recent advances regarding the expression and regulation of aquaporins in both clinical and experimental models of sepsis. We present an overview regarding AQP1, AQP2, AQP3, AQP4, AQP5, and AQP9 expression and regulation at the transcriptional, translational, and post-translation levels. The reviewed literature covers septic patients and experimental models of sepsis, including mice or rats exposed to lipopolysaccharide (LPS), mice or rats on which cecal-ligation and puncture (CLP) was performed, and human cells exposed to LPS. 

## 2. Aquaporin Expression in Clinical and Experimental Models of Sepsis

**Figure 2:**
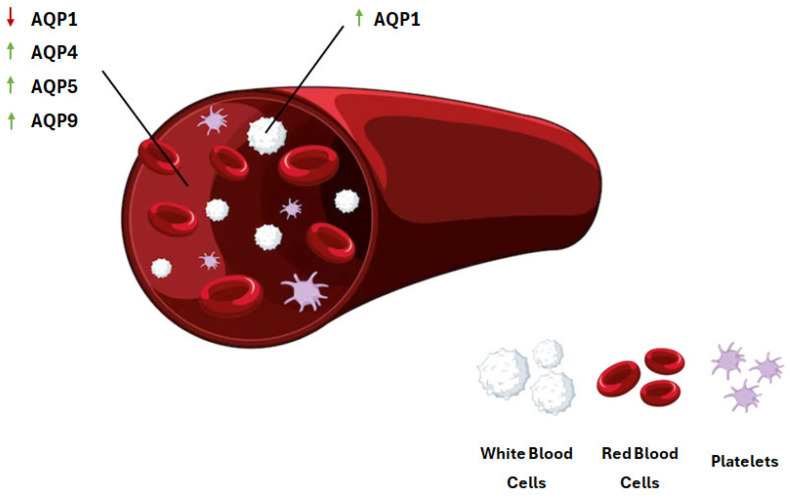
Dysregulation of aquaporins in clinical sepsis. AQP1 expression is upregulated in the leukocytes of critically ill septic patients, while serum levels of AQP1 are downregulated. AQP4, AQP5, and AQP9 expression are upregulated in the blood of septic patients. AQP, aquaporin. Green arrows depict upregulation of AQP expression and red arrows depict downregulation. AQP, aquaporin. This figure has been designed using assets from Freepik.com.

Few studies have investigated the expression of aquaporins in a clinical septic setting. The results of these studies have, however, demonstrated that the expression of aquaporins is dysregulated in sepsis, as well as other pathological conditions that arise due to sepsis development. In a clinical setting, our research group has demonstrated that leukocyte *AQP1* mRNA expression levels were significantly elevated in ICU patients who developed sepsis compared to non-septic ICU patients [10]. Matshushima and colleagues demonstrated that *AQP9* expression was significantly upregulated in the polymorphonuclear neutrophils (PMNs) of patients diagnosed with systemic inflammatory response syndrome (SIRS) [11]. In whole-blood samples of sepsis non-survivors, *AQP5* mRNA expression was shown to be increased in comparison to sepsis survivors [12]. In a cohort of twelve healthy human subjects, eight of whom were administered intravenous LPS, the *AQP9* mRNA levels in peripheral blood leukocytes were significantly elevated following LPS administration [13]. Recently, Xie and colleagues analyzed the expression of *AQP9*, among other genes, in the GSE32707 dataset. The dataset included mRNA expression data from whole-blood samples of healthy individuals and patients with acute respiratory distress syndrome (ARDS). ARDS is a life-threatening disorder, common in the setting of sepsis, comprised of many pathological attributes, including diffuse endothelial injury, increased capillary permeability, and hypoxemia. Furthermore, ARDS constitutes one of the leading causes of death in the ICU [14,15]. The initial results of these studies demonstrated an upregulation of *AQP9* mRNA expression in ARDS patients, although when a validation analysis was performed on a separate group of ARDS patients and healthy individuals, no significant changes in *AQP9* expression were detected [16]. Finally, AQP4 protein levels were found to be elevated in the blood samples of patients suffering from sepsis-associated encephalopathy (SAE) [17]. Table 1 presents an overview of human studies on the expression of AQPs in the setting of sepsis and related disorders. The dysregulation of aquaporin expression that occurs in sepsis is depicted in Figure 2.

As opposed to human studies, more data exist from studies performed on various experimental septic models. In PMNs isolated from healthy donors, stimulation with LPS resulted in an increase in AQP1 mRNA and protein expression [10]. Furthermore, da Silva and colleagues demonstrated that in human leukocytes, exposure to LPS resulted in elevated levels of *AQP9* mRNA. In addition, *AQP9* and *AQP3* mRNA upregulation was reported in LPS-exposed monocytes originally treated with phorbol myristate acetate (PMA) [20]. 

One of the most common complications of sepsis is lung injury. Aquaporin expression has been studied in several animal and cell models of sepsis-induced lung injury. Li et al. studied the expression of AQP1, AQP3, AQP4, and AQP5 in the lungs of mice exposed to LPS. They reported that protein expression of AQP1 and AQP5 decreased significantly at 4 and 8 h post-LPS exposure, while AQP3 and AQP4 protein levels remained unaltered [21]. Furthermore, AQP1 and AQP5 protein expression decreased in the alveoli of mice with LPS-induced lung injury [22]. Rump and colleagues demonstrated that in the same lung injury model, lung *AQP1* expression was downregulated, while the mRNA expression of *AQP5* remained unaltered [23]. Moreover, work from our research group has reported the expression patterns of aquaporins in several animal models of lung injury. Specifically, in LPS-exposed mice, mRNA expression and protein levels of AQP5 were found to be downregulated, while AQP1 expression remained unaffected. Interestingly, *AQP9* expression was found to increase significantly, although no changes in the protein levels were identified [24]. Additionally, in rats, both the mRNA and protein levels of AQP1 and AQP5 were found to decrease significantly in lung tissues following exposure to LPS [25]. The same results regarding AQP1 and AQP5 mRNA and protein expression were demonstrated in a CLP rat model by Guo and colleagues [26]. Furthermore, CLP-induced septic rats exhibited increased expression of both *AQP3* and *AQP4* mRNA in the pulmonary vasculature, while only the protein expression of AQP3 was found to be elevated [27]. 

The expression of aquaporins has also been investigated in experimental models of sepsis-induced acute kidney injury. Wang et al. examined the expression of several aquaporins in renal tubular epithelial cells following LPS exposure for 12 h. Among all the studied aquaporins, only *AQP1* and *AQP2* mRNA expression was altered, showing a significant decrease [28]. Additionally, Li et al. reported a difference between AQP1 mRNA and protein levels in the kidney tissues of LPS-induced rats. Specifically, *AQP1* expression in kidney tissues was decreased following injury, presenting a steady increase with time, while the AQP1 protein expression in the serum and kidney tissues was initially upregulated and diminished with time [29]. 

**Figure 3:**
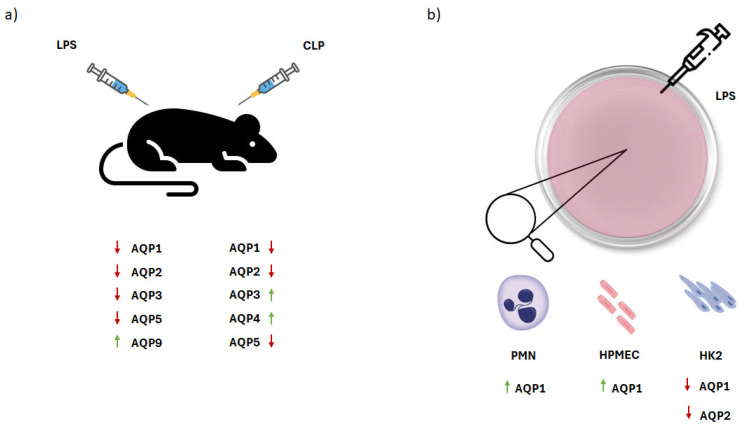
Dysregulation of aquaporins in experimental sepsis. (**a**) Aquaporin expression in animal experimental models following LPS treatment or CLP injection. In LPS-induced experimental models, AQP1, AQP2, AQP3, and AQP5 are downregulated, while AQP9 expression is upregulated. In CLP models, AQP1, AQP2, and AQP5 expression levels are downregulated, while the expression levels of AQP3 and AQP4 are upregulated. (**b**) Aquaporin expression in in vitro cell models. In PMNs and HPMECs, AQP1 expression is elevated, and in HK2 cells, AQP1 and AQP2 expression levels are downregulated. Green arrows depict upregulation of AQP expression and red arrows depict downregulation. AQP, aquaporin; CLP, cecal ligation and puncture; HK2, human kidney cells 2; HPMEC, human pulmonary microvascular endothelial cells; LPS lipopolysaccharide; PMNs, polymorphonuclear neutrophils. This figure has been designed using assets from Freepik.com.

Kidney AQP2 protein levels have been shown to decrease in both CLP- and LPS-induced septic rats [30,31]. In rats injected with LPS, Olesen and colleagues showed that following treatment, AQP2 protein levels were downregulated in the cortex and the inner stripe of the outer medulla, while no significant changes were observed in the inner medulla [32]. Moreover, Wang and colleagues demonstrated that in endotoxemia-induced mice, the protein expression of AQP2 and AQP3 decreased significantly in the kidneys following treatment with LPS [33]. Table 2 lists the experimental sepsis models that have been used to study the expression of AQPs, and Figure 3 summarizes the dysregulation of aquaporin expression that occurs in these models.

## 3. Aquaporin Long Non-Coding RNAs and Micro RNAs in Clinical and Experimental Models of Sepsis

Many long non-coding RNAs (lncRNAs) and micro RNAs (miRNAs) have been described as regulators of aquaporin expression in different conditions, including lung injury, acute kidney injury, cerebral ischemic reperfusion injury, and various cancer types. Recent endeavors have shed light on the involvement of aquaporin lncRNAs and miRNAs in the pathophysiology of sepsis. Long non-coding RNAs are non-protein coding transcripts consisting of more than 200 nucleotides that can regulate gene expression on epigenetic, transcriptional, and post-transcriptional levels. lncRNAs are of great importance in a plethora of biological processes, including chromosome modification, transcriptional regulation, and genomic imprinting [50]. On the other hand, microRNAs are small non-coding RNAs that are composed by an average of 22 nucleotides. The interaction of miRNAs with gene regions, mainly the 3′ untranslated region (3′ UTR), results in translational repression, although on certain occasions, gene activation has been reported [51,52]. As with lncRNAs, the involvement of miRNAs in many biological processes is well established. Most importantly, the pathophysiology of numerous human diseases has been connected to dysregulated expression of miRNAs [53].

In recent years, the involvement of both lncRNAs and miRNAs in the regulation of sepsis pathophysiology has been examined more rigorously, with members of both RNA groups serving as promising diagnostic markers and potential therapeutic targets [54]. Initially, Fang et al. demonstrated that in the setting of sepsis, the lncRNA H19 functions as an AQP1 competitive endogenous RNA (ceRNA) in regulating miR-874, which directly interacts with AQP1. The expression levels of *H19* and *AQP1* were found to be diminished in the sera of septic patients, while miR-874 levels were elevated, showing a negative relationship with both H19 and AQP1 [18]. 

miR-874 is a novel anti-cancer miRNA. Through the inhibition of gene expression, miR-874 retains a key role in numerous cellular mechanisms, including but not limited to apoptosis, cell proliferation, and migration [55]. Interestingly, in sepsis-induced ARDS, lncRNA H19 was shown to have great value as an early diagnostic tool. Specifically, the serum H19 levels of septic patients were decreased compared to healthy individuals, and thus could be potentially used as a tool for early sepsis diagnosis [56]. The long non-coding RNA cancer susceptibility candidate 2 (CASC2) is another lnRNA that has been demonstrated to regulate *AQP1* expression in lung carcinoma epithelial cells and lung injury mouse models via the miR-144-3p/AQP1 axis [34]. In humans, CASC2 levels have been found to be decreased in septic patients compared to healthy individuals and could be utilized as a biomarker for disease severity and mortality [57]. 

LncRNA-5657 is also of great importance in the development of ARDS. The expression levels of lncRNA-5657 were found elevated in the bronchoalveolar lavage fluid (BALF) cells of patients with sepsis-induced ARDS [58]. Although lnc-5657 has not been shown to regulate aquaporin expression in sepsis-induced ARDS, a link between lnc-5657 and AQP4 has been identified in sepsis-associated encephalopathy (SAE). The main characteristic of SAE is diffuse cerebral dysfunction induced by the systemic response to infection [59]. Finally, AQP1 has been shown to be regulated by miR-126-5p. Healthy individuals were found to exhibit elevated plasma levels of miR-126-5p compared to septic patients and sepsis-induced ARDS patients. Interestingly, patients suffering from sepsis-induced ARDS also demonstrated decreased miR-126-5p levels compared to septic patients [60].

In sepsis-induced acute kidney injury, *AQP2* expression has been shown to be regulated by miR-34b-5p. The serum levels of miR-34b-5p were found to be elevated in sepsis-induced acute kidney injury (AKI) patients. Additionally, the decreased AQP2 gene and protein expression found in human renal tubular epithelial cells (HK-2) treated with LPS were negatively regulated by miR-34b-5p, thus promoting apoptosis and an inflammatory response [45].

In experimental sepsis models, upregulation of the lncRNA H19 has been demonstrated in CLP rats with sepsis-induced lung injury, resulting in the reduction of both apoptosis and pulmonary inflammation. This prompted the authors to suggest its role as a possible therapeutic target [56]. The initial exposure of mice to LPS induced apoptosis and resulted in decreased expression of both lncRNA CASC2 and *AQP1*, while miR-144-3p expression increased. CASC2 overexpression reversed the effects of LPS on *AQP1* gene expression and reduced apoptosis by regulating miR-144-3p, which binds to the 3′ UTR of the *AQP1* gene [34]. The significance of lncRNA CASC2 in regulating expression levels during sepsis-induced lung injury has also been pinpointed in other studies. CASC2 has been shown to serve as a competing endogenous RNA of miR-27b and miR-152-3p, which downregulate transforming growth factor-β-activated kinase 1 binding protein 2 (TAB2) and pyruvate dehydrogenase kinase 4 (PDK4), respectively, in lung injury models [61,62]. In a murine LPS-induced acute lung injury model, overexpression of miR-126-5p in alveolar type II (ATII) cells was shown to ameliorate the reduction in AQP1 protein expression, which resulted from the LPS treatment [35]. In the lung tissue of a CLP-induced sepsis rat model, downregulation of lncRNA-5657 resulted in a reduction in lung inflammation caused by CLP [58]. Moreover, the elevated AQP4 protein levels found in cortical and hippocampal tissues of CLP-induced SAE mice could be reduced via the downregulation of lncRNA-5657 [17]. Finally, the neuronal degradation and necrosis found in the hippocampus of CLP sepsis-induced rats were attenuated following lncRNA-5657 silencing [63]. As in the case of ARDS, lncRNA-5657 downregulation repressed the progression of SAE. 

In an LPS-induced lung injury mouse model, the intravenous injection of a miR-34b-5p antagomir in vivo significantly inhibited miR-34b-5p upregulation, reduced inflammatory cytokine release, decreased alveolar epithelial cell apoptosis, attenuated lung inflammation, and improved survival. The authors suggested that the *AQP2* expression regulator miR-34b-5p may be a potential target for lung injury treatments [64]. 

AQP5 regulation by miRNAs has been examined in the lungs of an LPS-induced disseminated intravascular coagulation (DIC) rat model. DIC is defined as the systemic intravascular activation of coagulation that leads to fibrin formation and deposition in the microcirculation. DIC is a frequents result of sepsis [65]. Zhang et al. reported that following LPS exposure, the lung levels of AQP5 mRNA and protein expression decreased, while miR-96 and miR-330 expression levels were found elevated, suggesting that AQP5 is directly regulated by both miRNAs [48]. MicroRNA-96 is a member of the miR-183-96-182 cluster, playing a significant role in cell migration and tumor progression, while miR330 has been shown to be capable of acting as a suppressor or activator of numerous processes associated with the progression of malignancies [66,67].

## 4. Aquaporin Regulators in Experimental Models of Sepsis

A growing number of studies have explored the effects of various molecules on the regulation and function of aquaporins and how they may relate to the attenuation of conditions that arise due to sepsis manifestation. 

Many of these studies have focused on identifying molecules with prophylactic effects against sepsis-induced lung injury that act by regulating the expression of aquaporins. Several molecules with a regulatory effect on aquaporin 5 expression have been identified. Lipoxin A_4_ (LXA4) has been shown to alleviate sepsis-induced lung injury in both LPS-exposed mice and cecal ligation rat models [47,68]. Lipoxins are bioactive autacoid metabolites and are the byproducts of the reaction between arachidonic acid and lipoxygenase enzymes. Lipoxins form during inflammation and activate cellular pathways to elicit their anti-inflammatory role. Lipoxin A_4_ is naturally occurring and one of the two originally identified lipoxins [69,70]. More specifically, in the LPS-induced lung injury model, exposure to LPS originally resulted in decreased levels of AQP5 protein expression. Following treatment with lipoxin A_4_, AQP5 protein levels in the lung tissue of sepsis-induced mice increased significantly, which was possibly attributed to the reduction in p38 and c-Jun N-terminal kinase (JNK) phosphorylation. Hence, the authors concluded that LXA4 plays a protective role in LPS-induced lung injury by upregulating AQP5 expression, suggesting a potential new mechanism of LXA4 as an anti-inflammation therapy for the impairment of alveolar fluid transport in ALI [47].

AQP5 mRNA and protein expression can also be regulated by tanshinol. Tanshinol has been identified as the main active compound of *Salvia miltiorrhiza* Bunge, which is acknowledged by Chinese medicine as a treatment for cardiovascular diseases [71]. Sepsis-induced lung injury in rats following CLP resulted in diminished AQP5 mRNA and protein expression. Tanshinol treatment was able to reverse this effect and return AQP5 expression close to pre-treatment levels. Moreover, tanshinol attenuated the influx of tumor necrosis factor α (TNF-α) and interleukin-6 (IL-6) concentrations in rat lung tissue, exhibiting an anti-inflammatory role. It was concluded that tanshinol may upregulate the expression of AQP5 by inhibiting the inflammatory cytokines and the phosphorylation of p38, therefore protecting the lung tissue of rats with sepsis [49].

Fasudil, a selective Rho kinase (ROCK) inhibitor, has been demonstrated to impact AQP5 expression in a similar fashion to tanshinol. Fasudil pre-treatment and the subsequent exposure of mice to LPS resulting in lung injury, resulted in an upregulation of both AQP5 mRNA and protein levels, which were diminished in the LPS-exposed mice. LPS-exposed mice pre-treated with fasudil resulted in the downregulation of IL-6 in the lungs, the peripheral blood, and in the bronchoalveolar fluid (BALF). Hence, fasudil seemed to alleviate LPS-induced lung injury by restoring AQP5 expression, eliminating LPS-induced lung edema, and preventing LPS-induced pulmonary inflammation by inhibiting inflammation in the lungs [46].

More recent studies have generated data with regard to other molecules that can attenuate sepsis-induced lung injury by regulating AQP1 expression. A study by Liang and colleagues demonstrated the role of the myocyte-specific enhancer factor 2C (MEF2C), a transcription factor and member of the Mef2 family, in the suppression of sepsis-induced lung injury in rats. They demonstrated that CLP-induced lung injury resulted in a reduction in MEF2C mRNA and protein levels, whereas MEF2C treatment attenuated the progress of lung injury while simultaneously upregulating AQP1 mRNA and protein expression. Furthermore, the serum concentrations of TNF-α, IL-1β, and IL-6 were decreased, while the concentration of IL-10 was elevated following MEF2C treatment. More importantly, inhibition of AQP1 expression reversed the effects of MEF2C on lung injury suppression, suggesting a possible connection between MEF2C and AQP1 expression in the progression of lung injury [40]. 

Hypoxia-inducible factor 1α (HIF-1α) plays a significant role in the regulation of AQP1 expression. In human pulmonary microvascular endothelial cells (HPMECs) exposed to LPS, AQP1 mRNA and protein expression was elevated, as was its function, as was demonstrated by an increase of the cells’ volume. Interestingly, silencing of *HIF1A* expression in the LPS-induced cells attenuated AQP1 upregulation, thus indicating that *HIF1A* is a potential therapeutic target [41]. 

In addition to the above-mentioned studies, several molecules have been demonstrated to simultaneously impact both AQP1 and AQP5 expression levels in the presence of lung injury. The estrogen steroid hormone estradiol can inhibit processes integral to lung injury development. Pre-treatment with estradiol of mice exposed to LPS resulted in a significant upregulation of both AQP1 and AQP5 mRNA and protein levels compared to the untreated LPS-induced lung injury group. Increases in AQP1 and AQP5 expression were accompanied by reduced oxidative stress and inflammatory responses [36]. Research from the same group attributed a similar role of soy isoflavone to that of estradiol in the regulation of AQP1 and AQP5 expression. Initially, LPS exposure of rats resulted in decreased AQP1 and AQP5 mRNA and protein levels in the lung tissues. Pre-treatment of rats with increasing concentrations of soy isoflavone resulted in a dose-dependent upregulation of AQP1 and AQP5 mRNA and protein expression. Additionally, soy isoflavone was found to decrease TNF-α, IL-1β, and IL-6 levels, alleviating pulmonary edema and lung damage [37]. 

Another molecule connected to the upregulation of aquaporins is emodin, which is a naturally occurring anthraquinone derivative. Emodin can be isolated from *Rheum palmatum* or *Polygonam multiflorum*. It acts as a suppressor of various signaling pathways and has been shown to have anti-inflammatory properties in combination with other substances [72]. Cecal ligation puncture in rats decreased mRNA and protein expression levels of both AQP1 and AQP5. Pre-treatment of rats with emodin significantly increased both mRNA and protein expression levels at 12 h post-CLP. At the same timepoint, emodin was shown to suppress sepsis-induced pulmonary apoptosis and had a positive effect on mortality [26,73]. 

Finally, hydrogen-rich saline has been shown to reverse the downregulation of AQP1 and AQP5 mRNA and protein expression in an LPS-induced lung injury rat model. The investigators suggested that hydrogen-rich saline participates in the inhibition of p38 mitogen-activated protein kinase (MAPK) and JNK, thus resulting in an upregulation of aquaporin expression [38]. Another study demonstrated that hydrogen-rich saline could suppress lung injury in LPS-exposed septic rats via reduced apoptosis [74].

Two recent studies focused on the molecules regulating AQP3 and AQP4 expression in sepsis-induced lung injury. In both CLP-induced septic rats and LPS-treated pulmonary vein endothelial cells, AQP3 expression was significantly upregulated, and this correlated with increased pulmonary vascular permeability. Treatment with Ss-31, a novel antioxidant, resulted in decreased expression of AQP3 and pulmonary vascular permeability [27]. Furthermore, AQP4 expression has been reported to increase following sepsis-induced lung injury in LPS-exposed mice and CLP-rats [27,75]. In mice with LPS-induced lung injury, TGN-020 treatment, a selective AQP4 inhibitor, resulted in downregulation of AQP4, suppression of inflammatory cytokine production, and better survival rates [75]. 

As seen above, many studies have focused on sepsis-induced lung injury. However, studies exploring the possibility of various molecules in the regulation of aquaporins in sepsis-induced kidney injury also exist. One of the studied molecules is selenium, a trace element that mainly serves as an antioxidant and participates in combating inflammation [76,77]. Candan and colleagues explored the effects of selenium on rats with LPS-induced kidney injury. Specifically, selenium treatment resulted in an upregulation of AQP1 expression, which was initially decreased by LPS exposure [39]. Furthermore, Ozden and colleagues demonstrated the protective effect exerted by dexpanthenol in LPS-induced kidney injury. Dexpanthenol is a precursor of vitamin B, with proven anti-apoptotic and anti-inflammatory properties [78,79]. Interestingly, in rats exposed to LPS, treatment with dexpanthenol increased *AQP2* mRNA expression in kidney tissues through the silent information regulator 1 (SIRT1) signaling pathway [43]. Liu et al. demonstrated that the downregulation of AQP2 protein expression in rats with LPS-induced kidney injury could be attenuated by rhein treatment, an anthraquinone utilized in Chinese medicine with anti-inflammatory, antioxidant, and hepatoprotective properties [42,80]. Pre-treatment with propofol has been demonstrated to protect LPS-exposed rats with sepsis-induced kidney injury from further renal complications by preventing the downregulation of *AQP2* expression [44]. In the case of AQP9, recent studies have demonstrated that inhibition of its expression may present beneficial effects against sepsis progression. Pre-treatment with RG100204, a novel inhibitor of AQP9 expression, has been shown to attenuate cardiac and renal dysfunction and hepatocellular injury caused by CLP-induced sepsis in mice [81]. Interestingly, knockout of the *AQP9* gene in LPS-exposed mice has been demonstrated to increase survival rates, reduce the expression of the inflammatory transcription factor, nuclear factor kappa-light-chain-enhancer of activated B cells (NF-kB)-p65, and protect them from LPS-induced oxidative stress [82]. 

Table 3 lists the regulators of AQP expression and their effects on experimental sepsis progression.

## 5. The Role of Aquaporin Single-Nucleotide Polymorphisms in Clinical Sepsis

In recent years, single-nucleotide polymorphisms (SNPs) in aquaporin genes have been associated with numerous pathological conditions, presenting great clinical value. Specifically, the 1364 A/C polymorphism in the gene promoter region of *AQP5* has been extensively studied in sepsis. An initial study demonstrated that in patients with severe sepsis, 30-day survival was strongly associated with the presence of the 1364 A/C polymorphism. More specifically, 1364 A/C seemed to have a protective role, whereas carriers of the AA genotype were at a higher mortality risk [83]. Furthermore, the 1364 A/C polymorphism was strictly associated with the expression levels of *AQP5* and the migration of immune cells. *AQP5* mRNA expression was significantly greater in the blood of septic patients carrying the AA genotype compared to AC carriers. Additionally, neutrophils from AA donors demonstrated faster and greater migration compared to AC carriers [19]. These results could be attributed to a protective role of 1364 A/C in the reduction of *AQP5* expression, cell migration, and proliferation. The presence of the 1364 A/C polymorphism has been implicated in the development of major adverse kidney events in patients suffering from sepsis. Specifically, AC carriers were found to be less likely to develop major adverse kidney events within the first 30 days. Furthermore, they presented with significantly lower risk of death within 90 days compared to patients carrying the AA genotype [84]. 

The use of *AQP5* 1364 A/C as a survival prognostic factor has also been examined in ARDS patients. In a cohort of 136 patients suffering from ARDS, individuals carrying the *AQP5* AC genotype demonstrated greater 30-day survival rates compared to patients carrying the AA genotype. The results demonstrated that the *AQP5* 1364 A/C polymorphism could be used as a prognostic factor for 30-day survival in combination with other established predictors [85]. The same investigators demonstrated that in ARDS patients suffering from acute kidney injury (AKI), *AQP5* 1364 A/C was associated with the patients’ recovery rate. Specifically, individuals carrying the *AQP5* AC genotype presented with significantly higher AKI recovery rates on day 30 compared to AA carriers [86]. 

DNA methylation of the *AQP5* promoter region is central in the regulation of the gene’s expression. A hypomethylated state of the *AQP5* promoter, combined with increased binding of the specificity protein 1 (SP1) transcription factor, resulted in increased *AQP5* expression [87]. Apart from the increased *AQP5* expression discussed above, increased methylation of C (nt-937), which is located within the promoter region of *AQP5* and is a target of NF-kB, has been also shown in septic non-survivors compared to septic survivors. Additionally, methylation of the *AQP5* promoter site nt-937 could be used as a prognostic tool for 30-day mortality in septic patients [12]. Methylation of the *AQP5* promoter was examined in neutrophils, monocytes, and lymphocytes from healthy individuals and septic patients. Carries of the AC genotype exhibited increased methylation in both groups [88].

## 6. Conclusions

This review focused on describing recent advancements regarding the expression and regulation of aquaporins in sepsis and conditions induced by sepsis such as ARDS and AKI. We examined recently published studies on clinical sepsis and well-established experimental models of sepsis. Many studies have demonstrated the interplay between long non-coding RNAs and micro RNAs in the regulation of aquaporins and how this interaction affects sepsis progression. Furthermore, in recent years, a growing number of studies have focused on exploring the effects of novel molecules on the expression of aquaporins during sepsis and their potential as possible therapeutic approaches against sepsis development. AQP1 and AQP5 expression has been found to be downregulated by numerous investigators in experimental models of sepsis-induced ARDS. Many molecules have been found to be capable of restoring expression patterns to normal levels while simultaneously attenuating lung injury progression. AQP3 and AQP4 have been found to promote sepsis development, with their suppression proving to be beneficial for the experimental models. Moreover, AQP1 and AQP2 upregulation by various molecules in the setting of sepsis-induced AKI has been shown to suppress the disease’s development. Moreover, *AQP9* mRNA expression has been found to be elevated in human subjects injected with LPS as well as in a cohort of ARDS patients, while recent studies have demonstrated that inhibition of *AQP9* expression may protect patients from sepsis-induced complications. Finally, we explored the effects of SNPs on the expression of aquaporins and their importance as a clinical tool based on recent advancements. Future studies could explore whether molecules with proven effects in experimental models of sepsis present the same utility in a clinical setting. 

## Figures and Tables

**Table 1:** Aquaporin mRNA and protein expression in clinical sepsis.

Aquaporin	Findings	References
AQP1	mRNA upregulation in leukocytes of ICU septic patients	[10]
	mRNA downregulation in the serum of septic patients	[18]
AQP4	Protein upregulation in blood samples from SAE patients	[17]
AQP5	mRNA expression elevated in whole-blood samples of septic patients carrying the AA genotype of the 1364 A/C SNP compared to AC carriers	[19]
	mRNA expression in non-surviving septic patients in comparison to survivors	[12]
AQP9	mRNA upregulation in healthy humans injected with LPS	[13]
	mRNA upregulation in ARDS patients	[16]

AQP, aquaporin; ARDS, acute respiratory distress syndrome; ICU, intensive care unit; LPS, lipopolysaccharide; SAE, sepsis-associated encephalopathy; SNP, single-nucleotide polymorphism.

**Table 2:** Aquaporin mRNA and protein expression in experimental models of sepsis.

Aquaporin	Experimental Models	Findings	References
AQP1	LPS-exposed mice	Protein downregulation in the alveoli	[22]
	LPS-exposed mice	mRNA downregulated in the lungs	[23,34]
	LPS-exposed mice	Protein downregulation in the lungs	[21,35]
	LPS-exposed mice	mRNA and protein levels decreased in lung tissues	[36]
	LPS-exposed rats	mRNA and protein levels decreased in lung tissues	[25,37,38]
	LPS-exposed rats	mRNA decreased initially and presented with a steady increase in kidney tissue, serum protein increased initially and was downregulated in the serum and kidneys	[29]
	LPS-exposed rats	Protein levels decreased in the kidneys	[39]
	CLP-rats	mRNA and protein levels decreased in lung tissues	[26,40]
	LPS-exposed HPMECs	mRNA upregulation	[41]
	LPS-exposed HK2 cells	mRNA decrease	[28]
	LPS-exposed PMNs	mRNA and protein upregulation	[10]
AQP2	LPS-exposed mice	Protein downregulation in kidneys	[33]
	LPS-exposed rats	Protein downregulation in kidneys	[31,32,42]
	LPS-exposed rats	mRNA downregulation in kidneys	[43,44]
	CLP-rats	Protein downregulation in kidneys	[30]
	LPS-exposed HK2 cells	mRNA and protein decrease	[45]
	LPS-exposed HK2 cells	mRNA decrease	[28]
AQP3	LPS-exposed mice	Protein downregulation in kidneys	[33]
	CLP-rats	mRNA and protein upregulation in lung	[27]
	LPS-exposed PMA-treated monocytes	mRNA upregulation	[20]
AQP4	CLP-rats	mRNA upregulation in lungs	[27]
	CLP-rats	Protein increases in cortical and hippocampal tissues	[17]
AQP5	LPS-exposed mice	Protein expression decreased in the alveoli	[22]
	LPS-exposed mice	mRNA and protein levels decreased in lung tissues	[24,36,46]
	LPS-exposed mice	Protein expression decreased in lungs	[21,47]
	LPS-exposed rats	mRNA and protein levels decreased in lung tissues	[25,37,38,48]
	CLP-rats	mRNA and protein levels decreased in lung tissues	[26,49]
AQP9	LPS-exposed mice	mRNA upregulated in lung tissues	[24]
	LPS-exposed human leukocytes	mRNA upregulation	[20]
	LPS-exposed PMA-treated monocytes	mRNA upregulation	[20]

AQP, aquaporin; CLP, cecal ligation and puncture; HK2, human kidney cells 2; HPMECs, human pulmonary microvascular endothelial cells; LPS, lipopolysaccharide; PMA, phorbol myristate acetate; PMNs, polymorphonuclear neutrophils.

**Table 3:** Regulators of aquaporin expression and their effects on experimental sepsis progression.

Aquaporin	Experimental Models	Findings	References
AQP1	LPS-exposed mice	Estradiol treatment pre-LPS exposure upregulated AQP1 mRNA and protein levels, reducing oxidative stress and inflammatory responses	[36]
	LPS-exposed rats	Pre-treatment with increasing concentrations of soy isoflavone resulted in dose-dependent upregulation of AQP1 mRNA and protein, alleviating pulmonary edema and lung damage	[37]
	LPS-exposed rats	Hydrogen-rich saline reverses AQP1 mRNA and protein downregulation	[38]
	LPS-exposed rats	Selenium treatment resulted in the upregulation of AQP1 protein expression in the kidneys	[39]
	CLP rats	MEF2C treatment attenuated the progress of lung injury while upregulating AQP1 mRNA and protein expression	[40]
	CLP rats	Emodin pre-treatment significantly increased AQP1 mRNA and protein expression, suppressing sepsis-induced pulmonary apoptosis	[26]
	LPS-exposed HPMECs	Silencing *HIF1A* expression in LPS-induced cells attenuated AQP1 upregulation and regulated the cells’ volume increase	[41]
AQP2	LPS-exposed rats	Dexpanthenol increased *AQP2* mRNA expression in kidney tissues through the SIRT1 signaling pathway	[43]
	LPS-exposed rats	Rhein treatment attenuated the downregulation of AQP2 protein expression in the kidneys	[42]
	LPS-exposed rats	Propofol pre-treatment protected the rats from further kidney complications by restoring AQP2 mRNA levels	[44]
AQP3	CLP rats	Ss-31 treatment resulted in decreased protein expression of AQP3 and pulmonary vascular permeability	[27]
AQP4	LPS-exposed mice	TGN-020 treatment resulted in downregulation of AQP4, suppression of inflammatory cytokine production, and better survival rates	[75]
AQP5	LPS-exposed mice	Lipoxin A_4_ presents a protective role in lung injury by upregulating AQP5 protein expression in lung tissue	[47]
	LPS-exposed mice	Fasudil upregulates AQP5 mRNA and protein levels while eliminating LPS-induced lung edema and preventing LPS-induced pulmonary inflammation	[46]
	LPS-exposed mice	Estradiol treatment pre-LPS exposure upregulated AQP5 mRNA and protein levels, reducing oxidative stress and inflammatory responses	[36]
	LPS-exposed rats	Hydrogen-rich saline reverses AQP5 mRNA and protein downregulation	[38]
	LPS-exposed rats	Pre-treatment with increasing concentrations of soy isoflavone resulted in dose-dependent upregulation of AQP5 mRNA and protein, alleviating pulmonary edema and lung damage	[37]
	CLP rats	Tanshinol treatment reverses diminished AQP5 mRNA and protein levels while simultaneously inhibiting inflammatory cytokines and p38 phosphorylation	[49]
	CLP rats	Emodin pre-treatment significantly increased AQP5 mRNA and protein, suppressing sepsis-induced pulmonary apoptosis	[26]
AQP9	CLP mice	The novel inhibitor RG100204 had positive effects on renal and cardiac dysfunction	[81]

AQP, aquaporin; CLP, cecal ligation and puncture; *HIF1A*, hypoxia-inducible factor 1 alpha; HPMEC, human pulmonary microvascular endothelial cells; LPS, lipopolysaccharide; MEF2C, myocyte-specific enhancer factor 2C; SIRT1, silent information regulator 1; Ss-31, elamipretide.

## Data Availability

Not applicable.
